# Clinical observations and mechanistic insights of traditional Chinese medicine in the management of diabetic retinopathy

**DOI:** 10.1080/13880209.2024.2369292

**Published:** 2024-06-26

**Authors:** Jie Chen, Yadong Ni, Wenhui Yao, Xuansheng Ding

**Affiliations:** a School of Basic Medicine and Clinical Pharmacy, China Pharmaceutical University, Nanjing, China; b Precision Medicine Laboratory, School of Basic Medicine and Clinical Pharmacy, China Pharmaceutical University, Nanjing, China

**Keywords:** Chinese herb, inflammation, oxidative stress, neovascularization, blood-retinal barrier, neuroprotection, diabetic microvascular complication

## Abstract

**Context:**

Diabetic retinopathy (DR) is one of the leading causes of vision impairment and blindness among diabetic patients globally. Despite advancements in conventional treatments, the quest for more holistic approaches and fewer side effects persists. Traditional Chinese medicine (TCM) has been used for centuries in managing various diseases, including diabetes and its complications.

**Objective:**

This review evaluated the efficacy and underlying mechanisms of TCM in the management of DR, providing information on its potential integration with conventional treatment methods.

**Methods:**

A comprehensive literature review was conducted using PubMed, Web of Science, and the China National Knowledge Infrastructure (CNKI) with the search terms ‘traditional Chinese medicine’, ‘diabetic retinopathy’, ‘clinical efficacies’ and their combinations. Studies published before 2023 without language restriction were included, focusing on clinical trials and observational studies that assessed the effectiveness of TCM in DR treatment.

**Results:**

The review synthesized evidence of empirical traditional Chinese formulas, traditional Chinese patent medicines, and isolated phytochemicals on DR treatment. The key mechanisms identified included the reduction of oxidative stress, inflammation, and neovascularization, as well as the improvement in neurovascular functionality and integrity of the retinal blood barrier.

**Conclusions:**

TCM shows promising potential to manage DR. More large-scale, randomized controlled trials are recommended to validate these findings and facilitate the integration of TCM into mainstream DR treatment protocols.

## Introduction

Diabetes presents one of the most significant challenges facing global healthcare systems. The International Diabetes Federation (IDF) estimates that approximately 537 million adults worldwide are currently living with diabetes. This number is estimated to increase to 642 million by 2040, accounting for around 10% of the global adult population aged 20–79 years (Sun et al. 2022). Diabetic retinopathy (DR), a common complication of diabetes, often leads to severe vision impairment and is a leading cause of vision loss among diabetics in many countries (Dall et al. 2014; Mohamed et al. 2007). DR is characterized by damage to the small blood vessels in the retina, causing leakage or blockage of the retinal capillaries. The early stages of DR typically do not show warning signs, underlining its severity as a clinical consequence of diabetes. The importance of recognizing early warning signs needs to be emphasized, as untreated DR may lead to blindness.

DR is classified into non-proliferative diabetic retinopathy (NPDR) and proliferative diabetic retinopathy (PDR) (Wang and Lo 2018). Early manifestations include microaneurysms and intraretinal hemorrhages. Microvascular injury in DR can increase vascular permeability, leading to retinal edema and exudation. In its proliferative stage, DR may cause neovascularization within the optic disc, retina, iris, and eye angle, potentially leading to retinal detachment and neovascular glaucoma (Flaxel et al. 2020).

Pathological changes in early-stage DR primarily involve loss of pericytes and increased permeability of endothelial cells, altering the blood-retinal barrier (BRB). This disruption in the diabetic retina is central to loss of vascular integrity (Garner 1993; Rathnasamy et al. 2019) and is a hallmark of DR. The early stages are marked by the destruction of vascular endothelium and the breakdown of the BRB (Beltramo and Porta 2013; Mrugacz et al. 2021). DR pathology extends beyond vascular changes. Diabetes mellitus (DM) impairs retinal neuronal cell function before significant BRB changes occur. Early DR predominantly involves neurodegeneration, particularly ganglion cell degeneration. Therefore, synaptic neurodegeneration of retinal ganglion cells (RGCs) might be an initial step in DR etiology. Early lesion sites in DR also involve neurovascular units, comprising neurons, glial cells, and vascular cells, which are integral to the structural integrity of the BRB (Sun et al. 2023).

DR is a neurovascular disease that progresses over time, initially presenting neurological and vascular abnormalities, and in advanced stages, predominantly vascular abnormalities such as neovascularization (Rossino et al. 2019). Elevated blood glucose levels can induce retinal ischemia and hypoxia, damaging capillary endothelial cells. This damage triggers a reparative response, characterized by proliferation and thickening of the capillary basement membrane. Consequently, this leads to a cycle of capillary lumen narrowing, further exacerbating retinal ischemia and hypoxia. This cycle ultimately results in the development of microaneurysms, capillary occlusion, tissue hypoxia, papillary necrosis, and neovascularization (Heitzig et al. 2017; Lechner et al. 2017; Scanlon 2017). Early-stage DR (NPDR) typically lacks symptoms, while advanced-stage DR (PDR) can cause significant discomfort (Wei et al. 2022). Due to the insidious nature of early DR, detection often occurs at the PDR stage, when retinal damage is largely irreversible.

The clinical treatment of DR is mainly focused on the PDR stage, given the covert nature of early NPDR episodes (Wei et al. 2022). Proliferative DR is treated primarily with laser therapy and vitrectomy. Pharmacologically, anti-vascular endothelial growth factor (VEGF) drugs and steroids, mostly administered intravitreal injection, are the mainstays. However, these treatments can have side effects, including visual impairment or diabetic macular edema from surgical and laser treatments, intraocular inflammation, infection, retinal detachment from VEGF medications, and cataracts from steroid treatments in some cases (Heng et al. 2013). Traditional Chinese Medicine (TCM) has been used for thousands of years and has shown promise against DR, improving both NPDR and PDR outcomes (Nulahou et al. 2018; Wang, He, et al. 2019). Despite numerous publications in this evolving field, there remains a need for more current and systematic reviews. This review summarized the clinical efficacy of TCM in treating DR and its potential mechanisms, providing information for developing new anti-DR drugs.

## Materials and methods

### Study selection

The present review discusses traditional Chinese medicines (TCMs) in the management of diabetic retinopathy. With this objective, an extensive collection of scientific literature was examined by considering all highlighted research articles and reviews on the issue. Three main databases, PubMed, Web of Science, and the China National Knowledge Infrastructure (CNKI) were used as information sources by the inclusion of the search terms ‘traditional Chinese medicine’, ‘diabetic retinopathy’, ‘clinical efficacies’ and their combinations, 125 studies were included before 2023 without language restrictions.

Inclusion criteria: (1) Studies of empirical traditional Chinese formulas to improve diabetic retinopathy; (2) Studies of traditional Chinese patient medicines to improve diabetic retinopathy; (3) Studies of isolated phytochemicals to improve diabetic retinopathy.

Exclusion criteria: (1) Studies mainly focused on non-pharmaceutical therapy in Chinese medicine (such as acupuncture, moxibustion, and fumigating with herbs); (2) Studies with single surgical treatment without TCMs for improvement of diabetic retinopathy.

### Clinical efficacies of traditional Chinese medicines in DR treatment

#### Empirical traditional Chinese formulas

Empirical prescriptions, rooted in TCM and refined through extensive clinical practice, show promise for treating DR. Numerously empirical formulas, like the TCM Formula, have demonstrated significant efficacy in clinical settings. Sixty patients were randomly divided into two groups to examine the efficacy of the TCM Formula in treating DR. The control group (*n* = 30 cases) received routine treatments with the objective of controlling blood glucose, blood pressure and blood fat, and the observation group (*n* = 30 cases) received the TCM Formula in addition to the routine treatment. After 24 weeks of treatment, researchers confirmed that this formula had anti-inflammatory effects and showed improvements in DR patients in visual acuity, vascular leakage area, microvascular tumor count, and inflammatory factor levels (Yi et al. 2019).

The treatment of DR varies between its different stages. In the NPDR stage, formulas such as Jiang-Tang-Zeng-Ming Tang are commonly used. A study involving 84 NPDR patients, was divided into a control group (received 0.5 g calcium dobesilate capsules or epalrestat twice daily) and a treatment group (taken Jiang-Tang-Zeng-Ming Tang orally), which revealed improvements in the treatment group regarding visual acuity, fundus changes, fasting blood glucose, glycated hemoglobin, and overall efficacy over four months (*p* < 0.05) (Yi 2013). Similar positive outcomes were observed with the Zi-Yin-Yi-Qi-Tong-Luo Formula (Jia 2014) and the Tang-Wang Presentation (Jin et al. 2021) in treating Type 2 NPDR.

Additionally, Yi-Qi-Yang-Yin Decoction and Qing-Gan-Ming-Shi Tang have been proven to be effective in the treatment of mild to moderate NPDR, with the potential to impact distinct aspects of the condition. For example, these treatments have been shown to influence electroretinography and visual evoked potentials in mild NPDR, objective methods for assessing diabetic retinal function. Studies have reported changes in amplitude and implicit time in NPDR patients (Miura 2023; Tzekov and Arden 1999). With the Yi-Qi-Yang-Yin Decoction treatment, there was a notable shortening in the latency of the P50-wave, the N95-wave, and the P100-wave (*p* < 0.05), along with an increase in amplitude compared to the baseline (Zhang et al. 2019). Furthermore, combining Qing-Gan-Ming-Shi Tang with calcium dobesilate capsules improved clinical symptoms, vision, and severity of retinal microvascular disease in the fundus.

The deficiency of *Qi* and *Yin* syndrome, a common TCM classification in DM, often leads to stasis and is frequently observed in the middle phase of DM (Wang et al. 2020). The Yuan-Qi-Qu-Yu Formula which was effective for the NPDR stage has shown improvements in clinical symptoms, visual acuity, blood viscosity, and prognosis in NPDR patients with this syndrome (Nulahou et al. 2018). Additionally, this formula has been found to reduce retinal micro-aneurysms and alleviate retinal bleeding and exudation in PDR patients after vitrectomy, providing a novel approach to PDR treatment (Wang, He, et al. 2019). The Sheng-Qing-Jiang-Zhuo-Tong-Luo-Ming-Mu Formula in combination with conventional western medicine therapy was evaluated in DR treatment. This combination enhanced the total effective rate of improved retinal lesions (90% vs. 62%, *p* < 0.05) and visual acuity (82% vs. 57%, *p* < 0.05) compared to the control group (Shi et al. 2019). Similarly, Zhi-Xue-San-Yu-Ming-Mu Tang effectively treated *Qi-Yin* deficiency and meridian stasis syndrome, enhancing microcirculation and visual acuity (Cui et al. 2019).

The Yi-Qi-Tong-Luo Decoction obviously impacted early-stage PDR. In a study, 40 patients with stage IV DR were randomly divided into a control group (received standard blood sugar control) and a treatment group (given additional Yi-Qi-Tong-Luo Decoction). The treatment group exhibited a higher total effective rate (*p* < 0.05) and marked improvements from baseline to post-treatment (*p* < 0.05) (Zhang 2016). These findings, along with those of the Yuan-Qi-Qu-Yu formula which was mentioned above, underscore the substantial potential of TCM in treating PDR.

The severity of DR is evaluated based on traditional Chinese symptoms such as blurred vision, loss of vision, dryness of the eyes and dizziness, and the TCM syndrome score is assigned accordingly, as per the ‘Evidence-based guidelines for diagnosis and treatment of diabetic retinopathy in China’. The higher the score, the more severe the disease. Many TCM formulas, such as Da-Ming Drink (Sun and Jiang 2013; Wang, Zhang, et al. 2023), Xi-Shi Drink (Zhang et al. 2020) and Tian-Jiang-Xue-Shuan-Tong Pills (Dai et al. 2020), which have undergone dosage form modifications in clinical practice, were known to improve visual acuity and reduce TCM syndrome scores to effectively treat DR. Similarly, Shi-Qing Drink was effective in treating cataract patients with stasis-heat blocking collaterals in conjunction with NPDR, reducing macular retinal thickness (Zhang, Xu, et al. 2018). The Zheng-Yuan-Yun-Sheng Dripping Pills have shown notable efficacy in treating macular edema in DR patients (Guan et al. 2013). Both Mu-Xue-Kang Capsules and Pu-Ren Dan have demonstrated effectiveness in eliminating microangiomas and reducing the area of retinal exudation, as supported by research (Li, Feng, et al. 2017; Wang, Zhang, et al. 2019).

Table 1 shows the 19 clinical trials involving 17 different TCM formulas, all originating from the empirical formulas of modern Chinese medicine practitioners to treat DR. These formulas are grounded in TCM theories and knowledge about the compatibility of Chinese medicinal ingredients, demonstrating significant therapeutic efficacy in clinical applications.

**Table 1.** Clinical observation of Chinese empirical formulas for the treatment of diabetic retinopathy.

Name	Composition	Samples	Dosage/daily	Duration	DR stage	Hospital/center	Reference
The Traditional Chinese Medicine Formula	*Scutellaria baicalensis* Georgi (Lamiaceae), *Lycium barbarum* L., *Dioscorea opposita* Thunb., *Pueraria lobata* (Willd.) Ohwi, *Schisandra chinensis* (Turcz.) Baill., *Trichosanthes kirilowii* Maxim., *Eclipta prostrata* L. (Asteraceae), *Rehmannia glutinosa* (Gaertn) DC (Plantaginaceae), *Paeonia suffruticosa* Andr., *Dendrobium nobile* Lindl., *Paeonia lactiflora* Pall., *Rubia cordifolia* L., *Typha angustifolia* L., *Ligusticum chuanxiong* Hort.	30	Two times/day (six days continuously and stop one day)	Six months	DR	Wuhan No.9 Hospital	(Yi et al. 2019)
Jiang-Tang-Zeng-Ming Tang	*Scutellaria baicalensis* Georgi (Lamiaceae), *Pseudostellaria heterophylla* (Miq.) Pax, *Salvia miltiorrhiza* Bunge (Labiatae), *Rehmannia glutinosa* (Gaertn) DC (Plantaginaceae), *Conyza blinii* Lévl., *Cirsium japonicum* Fisch. ex DC., *Sparganium stoloniferum* Buch. Ham., *Curcuma phaeocaulis* VaL., *Poria cocos* (Schw.) Wolf, *Whitmania pigra* Whitman, *Glycyrrhiza uralensis* Fisch., *Panax notoginseng* (Burk.) F. H. Chen (Araliaceae), *Astragalus membranaceus* (Fisch.) Bge., *Angelica sinensis* (Oliv.) Diels (Apiaceae), *Atractylodes macrocephala* Koidz., *Leonurus cardiaca* L. (Lamiaceae)	42	One dose of Water Decoction/two times/day; 200 ml/time	Four months	NPDR	The People’s Hospital of Xinzhou District	(Yi 2013)
Zi-Yin-Yi-Qi-Tong-Luo Formula	*Astragalus membranaceus* (Fisch.) Bge., *Pueraria lobata* (Willd.) Ohwi, *Panax notoginseng* (Burk.) F. H. Chen (Araliaceae), *Lycium barbarum* L., *Typha angustifolia* L., *Leonurus cardiaca* L. (Lamiaceae), *Rehmannia glutinosa* (Gaertn) DC (Plantaginaceae), *Cassia obtusifolia* L. (Leguminosae), *Angelica sinensis* (Oliv.) Diels (Apiaceae), *Whitmania pigra* Whitman, *Pheretima aspergillum* (E.Perrier), *Dioscorea opposita* Thunb.	51	One dose of Water Decoction/two times/day; 200 ml/time	Six months	NPDR	Hebei Wanquan County Hospital of Traditional Chinese Medicine	(Jia 2014)
Yi-Qi-Yang-Yin Decotion	*Dendrobium nobile* Lindl., *Salvia miltiorrhiza* Bunge (Labiatae), *Pueraria lobata* (Willd.) Ohwi, *Anemarrhena asphodeloides* Bge., *Astragalus membranaceus* (Fisch.) Bge., *Rehmannia glutinosa* (Gaertn) DC (Plantaginaceae), *Rheum palmatum* L., *Glycyrrhiza uralensis* Fisch.	26	One dose of Water Decoction/two times/day; 200 mL/time	Three months	Mild to moderate NPDR (*Qi*-*Yin* deficiency and meridian stasis syndrome)	Shanghai Hospital of integrated Traditional Chinese and Western Medicin	(Zhang et al. 2019)
Tang-Wang Presentation (TWP)	*Astragalus sinicus* L., *Salvia miltiorrhiza* Bunge (Labiatae), Typha orientalis C. Presl, *Cassia obtusifolia* L. (Leguminosae), *Iris japonica* Thunb.	192	One dose/two times/day	One year	NPDR	Guang’anmen Hospital of China Academy of Chinese Medical Sciences; The First Affiliated Hospital of Anhui University of Traditional Chinese Medicine; Hubei Hospital of Traditional Chinese Medicine; Zhengzhou City Hospital of Traditional Chinese Medicine; Baodin City Hospital of Traditional Chinese Medicine; zi bo wanjie cancer hospital; Shijiazhuang City Hospital of Traditional Chinese Medicine; Zouping Country Hospital of Traditional Chinese Medicine	(Jin et al. 2021)
Qing-Gan-Ming-Shi Tang	*Conyza blinii* Lévl., *Panax notoginseng* (Burk.) F. H. Chen (Araliaceae), *Rehmannia glutinosa* (Gaertn) DC (Plantaginaceae), *Paeonia lactiflora* Pall., *Chrysanthemum morifolium* Ramat., *Cyathula officinalis* Kuan, Ophicalcitym, *Rubia cordifolia* L., *Glycyrrhiza uralensis* Fisch., Bupleurum chinense DC., *Cassia obtusifolia* L. (Leguminosae), *Curcuma wenyujin* Y. H. Chen et C. Ling	48	One dose of Water Decoction/two times/day	Six months	Mild to moderate NPDR	Cangzhou Hospital of integrated Traditional Chinese and Western Medicin	(Wang, Wu, et al. 2023)
Yuan-Qi-Qu-Yu Formula	*Astragalus membranaceus* (Fisch.) Bge., *Salvia miltiorrhiza* Bunge (Labiatae), *Ligustrum lucidum* Ait. (Oleaceae), *Scrophularia ningpoensis* Hemsl., *Sophora japonica* L., *Acorus tatarinowii* Schott, *Panax notoginseng* (Burk.) F. H. Chen (Araliaceae), *Ligusticum chuanxiong* Hort., *Leonurus cardiaca* L. (Lamiaceae)	34	One dose of Water Decoction/two times/day	Three months	NPDR (*Qi*-*Yin* deficiency syndrome)	Hospital of Chinese Medicine Affiliated to Xinjiang Medical University	(Wang, Yao, et al. 2019)
		35	One dose/two times/day	Two months	PDR	The TCM Hospital of Xinjiang Autonomous Region	(Nulahou et al. 2018)
Sheng-Qing-Jiang-Zhuo-Tong-Luo-Ming-Mu Formula	*Astragalus membranaceus* (Fisch.) Bge., *Atractylodes lancea* (Thunb.) DC., *Lycopus lucidus* Turcz. var. hirtus Regel, *Leonurus cardiaca* L. (Lamiaceae), *Crataegus pinnatifida* Bge., *Coptis chinensis* Franch., *Rheum palmatum* L., *Panax notoginseng* (Burk.) F. H. Chen (Araliaceae), *Citrus aurantium* L., *Magnolia officinalis* Rehd.et Wils., *Cassia obtusifolia* L. (Leguminosae), *Alisma orientale* (Sam.) Juzep., *Trichosanthes kirilowii* Maxim., *Buddleja officinalis* Maxim., *Cinnamomum cassia* Presl, *Scrophularia ningpoensis* Hemsl., *Dioscorea opposita* Thunb., *Salvia miltiorrhiza* Bunge (Labiatae)	100	Two times/day; 200 mL/time	Two months	NPDR (*Qi*-*Yin* deficiency and meridian stasis syndrome)	Fengrun District People’s Hospital	(Shi et al. 2019)
Zhi-Xue-San-Yu-Ming-Mu Tang	*Astragalus membranaceus* (Fisch.) Bge., *Anemarrhena asphodeloides* Bge., *Rehmannia glutinosa* (Gaertn) DC (Plantaginaceae), *Phellodendron chinense* Schneid., *Dioscorea opposita* Thunb., *Poria cocos* (Schw.) Wolf, *Alisma orientale* (Sam.) Juzep., *Trichosanthes kirilowii* Maxim., *Pueraria lobata* (Willd.) Ohwi, *Nelumbo nucifera* Gaertn., *Cryptotympana pustulata* Fabricius, *Equisetum hyemale* L., *Panax notoginseng* (Burk.) F. H. Chen (Araliaceae)	120	One dose of Water Decoction/two times/day	Three months	NPDR (*Qi*-*Yin* deficiency and meridian stasis syndrome)	Hebi Hospital of Traditional Chinese Medicine; Beijing Tongren Hospital; Beijing Huangsi Cosmetic Surgery Hospital	(Cui et al. 2019)
Yi-Qi-Tong-Luo Decoction	*Astragalus membranaceus* (Fisch.) Bge., *Salvia miltiorrhiza* Bunge (Labiatae), *Polygonatum kingianum* Coll.et Hemsl., *Dioscorea opposita* Thunb., *Typha angustifolia* L., *Panax notoginseng* (Burk.) F. H. Chen (Araliaceae), *Buthus martensii* Karsch	20	One dose of Water Decoction/two times/day; 200 mL/time	45 days	PDR (Stage IV)	Wuji Country Hospital	(Zhang 2016)
Da-Ming Drink	*Panax notoginseng* (Burk.) F. H. Chen (Araliaceae), *Typha angustifolia* L., *Polygonatum kingianum* Coll.et Hemsl., *Ligustrum lucidum* Ait. (Oleaceae), *Ligusticum chuanxiong* Hort., *Astragalus membranaceus* (Fisch.) Bge., *Bupleurum chinense* DC.	30 (eyes)	Two times/day; 150 mL/time	Three months	NPDR (*Qi*-*Yin* deficiency and blood stasis syndrome);	First Affilliated Hospital, Heilongjiang University of Chinese medicine	(Sun and Jiang 2013)
		45	Two times/day; 150 mL/time	One month	NPDR (*Qi*-*Yin* deficiency and blood stasis syndrome)	Zhoukou Eye Hospital	(Wang, Sun, et al. 2023)
Xi-Shi Drink	*Angelica sinensis* (Oliv.) Diels (Apiaceae), *Astragalus membranaceus* (Fisch.) Bge., *Panax notoginseng* (Burk.) F. H. Chen (Araliaceae)	79	Three times/day; 10 mL/time	Three months	NPDR (Blood stasis blocking collaterals syndrome)	The First Affiliated Hospital of Xiamen University	(Zhang et al. 2020)
Tian-Jiang-Xue-Shuan-Tong Pills	*Gastrodia elata* Bl., *Pheretima aspergillum* (E.Perrier), *Ligusticum chuanxiong* Hort., *Agkstrodon halys* (Pallas), *Astragalus membranaceus* (Fisch.) Bge., *Salvia miltiorrhiza* Bunge (Labiatae), *Paeonia lactiflora* Pall., *Acorus tatarinowii* Schott, *Crataegus pinnatifida* Bge., *Whitmania pigra* Whitman, *Dalbergia odorifera* T. Chen (Fabaceae)	35	Two times/day; 2 pills/time	Four months	NPDR (Blood stasis blocking collaterals syndrome)	Beichen District Hospital of Traditional Chinese Medicine; Community Health Service Center of Xiditou Town in Beichen District; Tianjin Eye Hospital	(Dai et al. 2020)
Shi-Qing Drink	*Chrysanthemum morifolium* Ramat., *Paeonia suffruticosa* Andr., *Lycium barbarum* L., *Scutellaria baicalensis* Georgi (Lamiaceae), *Rehmannia glutinosa* (Gaertn) DC (Plantaginaceae), *Lilium lancifolium* Thunb., *Dendrobium nobile* Lindl., *Dioscorea opposita* Thunb., *Platycodon grandiflorum* (Jacq.) A. DC. (Campanulaceae), *Eclipta prostrata* L. (Asteraceae), *Ligustrum lucidum* Ait. (Oleaceae)	30	Two times/day; 15 g/time	One month	Cataract with NPDR (Stasis-heat blocking collaterals syndrome)	Dongzhimen Hospital Affiliated to Beijing University of Chinese Medicine	(Zhang, Xu, Zhao, et al. 2018)
Zheng-Yuan-Yun-Sheng Dripping Pills	*Angelica sinensis* (Oliv.) Diels (Apiaceae), *Ligusticum chuanxiong* Hort., *Prunus persica* (L.) Batsch, *Carthamus tinctorius* L. (Asteraceae), *Achyranthes bidentata* Blume (Amaranthaceae), *Platycodon grandiflorum* (Jacq.) A. DC. (Campanulaceae), *Rehmannia glutinosa* (Gaertn) DC (Plantaginaceae)	20	Three times/day; 5 dripping pills/time	Three months	Diabetic macular edema	Jilin Province People’s Hospital	(Guan et al. 2013)
Pu-Ren Dan (PRD)	*Momordica charantia* L. (Cucurbitaceae), *Panax ginseng* C. A. Meyer (Araliaceae), *Salvia miltiorrhiza* Bunge (Labiatae), *Pueraria lobata* (Willd.) Ohwi, *Polygonum multiflorum* Thunb., *Whitmania pigra* Whitman	24	Two times/day; 9 g/time	Three months	NPDR	Affiliated Hospital of Chengde Medical College	(Wang, Zhang, et al. 2019)
Mu-Xue-Kang Capsules	*Manis pentadactyla* L., *Panax notoginseng* (Burk.) F. H. Chen (Araliaceae), *Pteria martensii* (Dunker)	121	Three times/day; 3 capsules/time	Six months	NPDR (Stage I and II)	Hebei Eye Hospital	(Li, Feng, et al. 2017)

#### Traditional Chinese patent medicines

Traditional Chinese patent medicines, standardized in preparation, have transitioned from empirical formulas to commercially available drugs for treating DR. These drugs, characterized by fixed formulation ingredients and doses, are used alone or in combination with other medications for DR treatment. This section reviewed eight trials involving five medicines which were approved by the National Medical Products Administration (NMPA): Qi-Ming Granules, Compound Danshen Dripping Pills (CDDPs), Tong-Luo-Ming-Mu Capsules, Liu-Wei-Di-Huang Pills, and Ginkgo Leaf Tablets (Table 2).

**Table 2.** Clinical observation of traditional Chinese patent medicines in diabetic retinopathy treatment.

Name	Composition	manufacturer	Dosage/daily	Samples	Duration	Hospital/center	Reference
Qi-Ming Granules	*Radix Astragali Mongolici, Radix Puerariae Lobatae, Radix Rehmanniae, Fructus Lycii, Semen Cassiae Obtusifoliae, Fructus Leonuri Japonici, Pollen Typhae, Hirudo*	Zhejiang Wansheng Pharmaceutical Co., Ltd.	Three times/day; 4.5 g each time	132	Three months	The Affiliated Hospital of Chengdu University of Traditional Chinese Medicine; The Dongzhimen Hospital Affiliated with Beijing University of Chinese Medicine; China-Japan Friendship Hospital; The Wangjing Hospital Affiliated with the China Academy of Chinese Medical Sciences; The First Affiliated Hospital of Xiamen University; Guangdong Province Traditional Chinese Medical Hospital; Ningxia People’s Hospital; The People’s Hospital of the Guangxi Zhuang Autonomous Region	(Huo et al. 2022)
			Three times/day; 4.5 g each time	47	Three months	Xinyang Maternal and Child Health Care Hospital	(Chen 2023)
Compound Danshen Dripping Pills (CDDP)	*Radix Salviae Miltiorrhizae, Radix Notoginseng, borneol*	Tasly Pharmaceutical Group Co., Ltd.	270, 540, 810 mg /three times/day	130	Six months	Guang’anmen Hospital, China Academy of Chinese Medical Sciences; China-Japan Friendship Hospital; The Affiliated Hospital to Changchun University of Chinese Medicine; The Affiliated Hospital to Chengdu University of Chinese Medicine; Jiangsu Province Hospital of Traditional Chinese Medicine; The Affiliated Union Hospital to Tongji Medical College of Huazhong University of Science and Technology; First Affiliated Hospital of Third Military Medical University; Wuxi Number 2 People׳s Hospital; Tang Center for Herbal Medicine Research, Pritzker School of Medicine	(Lian et al. 2015)
			405 mg/three times/day	28	Three months	China-Japan Friendship Hospital	(Luo et al. 2015)
Tong-Luo- Ming-Mu Capsules	*Paeonia lactiflora* Pall.*, Ginkgo biloba* Linn (Ginkgoaceae)*, Typha angustifolia* L.*, Panax notoginseng* (Burk.) F. H. Chen (Araliaceae)*, Rheum palmatum* L.*, Astragalus membranaceus* (Fisch.) Bge.*, Ligustrum lucidum* Ait. (Oleaceae), *Eclipta prostrata* L. (Asteraceae)*, Pueraria thomsonii* Benth.*, Rehmannia glutinosa* (Gaertn) DC (Plantaginaceae)*, Cassia obtusifolia* L. (Leguminosae), *Pheretima aspergillum* (E.Perrier)	Shijiazhuang Yiling Pharmaceutical Co., Ltd.	Three times/day; 4 capsules/time	293	Three months	Guang’anmen Hospital, China Academy of Chinese Medical Sciences; Dongfang Hospital of Beijing University of Chinese Medicine; Affiliated Hospital of Changchun University of TCM; The First Affiliated Hospital of Hunan University of TCM	(Ren et al. 2024)
			Three times/day; 4 capsules/time	69	Three months	The First Hospital of Hebei Medical University	(Zhang et al. 2013)
Liu-Wei-Di-Huang Pills and Ginkgo Leaf Tablets	LWDHP: *Rehmannia glutinosa* (Gaertn) DC (Plantaginaceae)*, Cornus officinalis* Sieb. et Zucc.*, Dioscorea opposita* Thunb.*, Alisma orientale* (Sam.) Juzep.*, Paeonia suffruticosa* Andr.*, Poria cocos* (Schw.) Wolf; GLT: *Ginkgo biloba* Linn (Ginkgoaceae)	LWDHP: Wanxi Pharmaceutical Co., Ltd.; GLT: Yangtze River Pharmaceutical Co., Ltd.	LWDHP: Three times/day; 8 pills each time; GLT: Three times/day; 2 tablets/time	70	Three years	Jiangsu Province Hospital of Traditional Chinese Medicine	(Zhao et al. 2016)
			LWDHP: Three times/day; 8 pills each time; GLT: Three times/day; 2 tablets/time	41	Two years	The Second People’s Hospital of Yongkang City	(Wu 2017)

Qi-Ming Granules and CDDPs were recommended in the ‘Guidelines for the Prevention and Treatment of Type 2 Diabetes in China (2020)’ for the NPDR stage. Qi-Ming Granules (Zhejiang Wansheng Pharmaceutical Co., Ltd., Batch number: Z20090036) have been validated in numerous clinical trials for effectively treating NPDR. A multi-centered, randomized, parallel-controlled clinical trial involving 132 DR patients demonstrated reductions in the retinal hemorrhage area (RHA) (*p* < 0.01), retinal exudation area (REA) (*p* < 0.01), HbA1c (*p* < 0.05), and TCM syndrome score (*p* < 0.001) after 12 weeks of treatment with Qi-Ming Granules, compared to the control group. Additionally, the safety of Qi-Ming Granules for NPDR treatment was confirmed through the absence of significant adverse events during the trial (Huo et al. 2022). Another study showed that the combination of Qi-Ming Granules and calcium dobesilate capsules resulted in a 95.74% clinical effective rate, higher than the 79.07% rate observed in patients treated with calcium dobesilate capsules alone (*p* < 0.05). Furthermore, this combination therapy led to reductions in RHA, REA, and the number of microaneurysms, attributed to its preventive effects against oxidative stress and neovascularization. Moreover, only four subjects experienced minor discomfort, and there was no significant difference in the incidence of side effects compared with the control group which received calcium dobesilate capsules. These findings suggested that Qi-Ming Granules were safe (Chen 2023).

CDDPs which were also recommended for DR treatment, were produced by Tasly Pharmaceutical Group Co., Ltd. A randomized, double-blind, placebo-controlled, multi-centered clinical trial assessed the efficacy of CDDPs through changes in fluorescence fundus angiography (FFA) and fundoscopic examination parameters over 24 weeks. The percentage of ‘Excellent’ and ‘Effective’ results in FFA was higher in the CDDPs groups when compared to the placebo group (high-dose: 74.42%; mid-dose: 76.75% vs. placebo: 28.27%, *p* < 0.001). Furthermore, the ‘Excellent’ and ‘Effective’ rates of fundoscopic examination were also higher in the CDDPs groups (*p* < 0.001) (Lian et al. 2015). Another study compared the effectiveness of CDDPs with calcium dobesilate capsules in a randomized, double-blind trial. Although there was no significant difference between the two medicines, all measured indices (best corrected visual acuity, mean visual field defect, fundus hemorrhage area, number of microaneurysms, fluorescent leakage area, and capillary non-perfusion area) improved (*p* < 0.05) (Luo et al. 2015).

Tong-Luo-Ming-Mu Capsules, manufactured by Shijiazhuang Yiling Pharmaceutical Co., Ltd., also known as Qi-Huang-Ming-Mu Capsules, received approval from NMPA in 2023. This capsule was indicated for type 2 NPDR with blood stasis, collateral obstruction, and *Qi*-*Yin* deficiency syndrome, offering a new treatment option for DR patients. A randomized, double-blind, positive-control, multi-centered Phase III clinical trial showed superior improvements in comprehensive curative effect (*p* < 0.01), TCM syndrome efficacy (*p* < 0.01), visual acuity (*p* < 0.01), and fundus changes (*p* < 0.05) when compared to calcium dobesilate capsules (Ren et al. 2024). Comparable therapeutic benefits were also observed in another study. It was noteworthy that only one case of appetite loss was observed in the Tong-Luo-Ming-Mu Capsules group, and the incidence of adverse reactions was 1.45%, which was lower than the calcium dobesilate capsules (12.31%) (Zhang et al. 2013).

Liu-Wei-Di-Huang Pills (Batch number: Z41022128), produced by Wanxi Pharmaceutical Co., Ltd., and Ginkgo Leaf Tablets (Batch number: Z20027949), containing ginkgo leaf extract and manufactured by Yangtze River Pharmaceutical Co., Ltd., have been studied in combination to prevent and treat DR. One hundred and forty outpatients with type 2 diabetes mellitus were randomly divided into the treatment and the control group. Both groups were given basal therapy to which the treatment group was supplemented with Liu-Wei-Di-Huang Pills and Ginkgo Leaf Tablets, while the control group was given the corresponding placebo. This crucial 3-year clinical study supported its effectiveness by reduced DR prevalence (8.5% vs. 25.0%, *p* < 0.05) (Zhao et al. 2016). A further 41 cases demonstrated that patients with type 2 diabetes mellitus who were treated with Liu-Wei-Di-Huang Pills combined with Ginkgo Leaf Tablets exhibited effective control of the rate of retinopathy, the rate of progression, and improvement in the rate of remission, with high drug safety (Wu 2017).

In conclusion, traditional Chinese patent medicines demonstrate remarkable efficacy in treating DR and are associated with minimal drug-related adverse effects. This safety profile positions these drugs as a potentially preferred first-line pharmacological option for DR management. Concurrently, the strategy to identify new therapeutic indications for existing drugs represents an innovative approach to the development of DR treatments.

#### Isolated phytochemicals

With the advancement of TCM research, an increasing number of TCM extracts and isolated phytochemicals have shown a high activity against DR. However, there have been relatively few clinical trials with these herbal monomers. To date, only two clinical studies have been conducted, focusing on improving the hemodynamics and disease state of DR.

A study investigated the effects of leaf extract (Egb 761) of *Ginkgo biloba* L. (Ginkgoaceae) on abnormal hemorheological parameters in diabetic patients. The study, conducted from March to August 2002, involved 25 DM patients 54 to 64 years old. The patients received a daily dose of 240 mg of Egb 761 (three times daily with two film-coated tablets, each containing 40 mg of Egb 761), and baseline hemorheological characteristics were measured and re-evaluated after three months. The study found that Egb 761 administration led to a decrease in the internal and plasma viscosity of erythrocytes, and a reduction in malondialdehyde levels in erythrocyte membranes (4.89 ± 0.07 vs. 3.97 ± 0.14, *p* < 0.05), indicating an improvement in lipid peroxidative stress. The evaluation of erythrocyte deformability, conducted using the flow resistance (*β*) model, demonstrated an improvement. Additionally, evaluations of peripheral circulation in type 2 DM patients, including retinal capillary blood flow velocity and blood oxygen transport efficiency, demonstrated increases after treatment. In summary, Egb 761 therapy improved peripheral circulation disorders, positively impacting blood circulation and reducing the incidence of retinopathy (Huang et al. 2004).

In another study, 30 patients were randomly divided into two groups to examine the efficacy of puerarin (400 mg/day, intravenously) in treating DR. The treatment group received puerarin, while the control group was administered Mikebao (500 μg/day, intramuscularly). After 6 weeks of puerarin treatment, some improvements were observed in the erythrocyte aggregation index, whole blood viscosity rate, plasma viscosity rate, fibrinogen, and erythrocyte sedimentation rate (*p* < 0.05). Furthermore, parameters such as the maximum systolic velocity of the central retinal artery, end-diastolic volume, acceleration, and central retinal venous reflux velocity were improved (*p* < 0.05). In particular, naked eye vision also improved in the treatment group compared to the control group (*p* < 0.01). Therefore, puerarin reduced blood viscosity, improved microcirculation, and played a beneficial role in treating diabetic retinopathy (Ren et al. 2000).

Although isolated phytochemicals, with their more defined compositions, have significant potential for new drug research and development, clinical research on these compounds remains limited and warrants further exploration.

### Preclinical observation for DR treatment

Preclinical studies have not only elucidated the therapeutic effects of TCMs on DR but also explored their mechanisms of action, providing theoretical support for the transition from in-hospital formulations to novel drug therapies.

The Qi-Huang-Yi-Shen Formula, based on classical formulations and clinical experience (Batch number: CN 104645132A), was found to effectively prevent retinal epithelial-mesenchymal transformation (EMT) in diabetic rats (Yuan et al. 2023). Xue-Shuan-Tong Injection (lyophilized) by Wuzhou Pharmaceutical Co., Ltd. (Batch number: Z45021769) which was recognized in the China Pharmacopoeia (2015), demonstrated protective effects against DR, probably by inhibiting inflammation and reducing advanced glycation end products (AGE) production, suggesting its potential as a promising DR treatment (Li et al. 2020). Dan-Hong Injection (DHI), from Jinan Buchang Pharmaceutical Co. Ltd. (Batch number: Z20026866), primarily for coronary heart disease, also showed satisfactory effects on DR. The protective functions of DHI in DR were associated with improved glycolysis and insulin sensitivity (Liu et al. 2015).

Several non-patented drugs, including empirical formulas and in-hospital preparations, have also shown effectiveness in DR treatment. Si-Miao-Yong-An Decoction (SMYAD), dating back to the Qing dynasty, was reported to relieve BRB dysfunction, reduce retinal inflammation, and diminish retinal angiogenesis in animal studies (Du et al. 2021). Pu-Ren Dan proved beneficial in DR treatment by preventing neurovascular dysfunction by inhibiting excessive endoplasmic reticulum stress and apoptosis in laboratory animals (Dai et al. 2016; Jia et al. 2015; Zhang et al. 2016). Tang-Luo-Tong, an in-hospital preparation, also alleviated DR symptoms (Wei et al. 2014).

In recent years, many potent phytochemicals have been extracted from Chinese herbs to combat DR. GRg1, a steroidal saponin from *Panax ginseng* C.A. Meyer (Araliaceae), reduced cell apoptosis in the ganglion cell layer and the inner nuclear layer (Gao et al. 2020; Li, Wang, et al. 2013). Andrographolide (Andro), the main compound of *Andrographis paniculata* Wall. ex Nees (Acanthaceae), was used in a study to treat NPDR and PDR in mice. Andro effectively prevented retinal vessel proliferation in PDR mice (*p* < 0.001) and inhibited vessel leakage and inflammation in NPDR mice (Yu, Lu, et al. 2015). The protective properties of TPP, a component of *Typha angustifolia* L. (Thyphaceae), were observed in rat models of DR, probably due to its anti-inflammatory effects and improved blood circulation (Chen et al. 2017; Lei et al. 2018).

These studies highlighted the significant therapeutic value of TCMs in treating DR. As research continues to unravel the pharmacological mechanisms of TCMs, more opportunities arise for their development into new drug therapies.

### Biological mechanisms involved in the protective effects of Chinese medicines against DR

**Figure 1.**
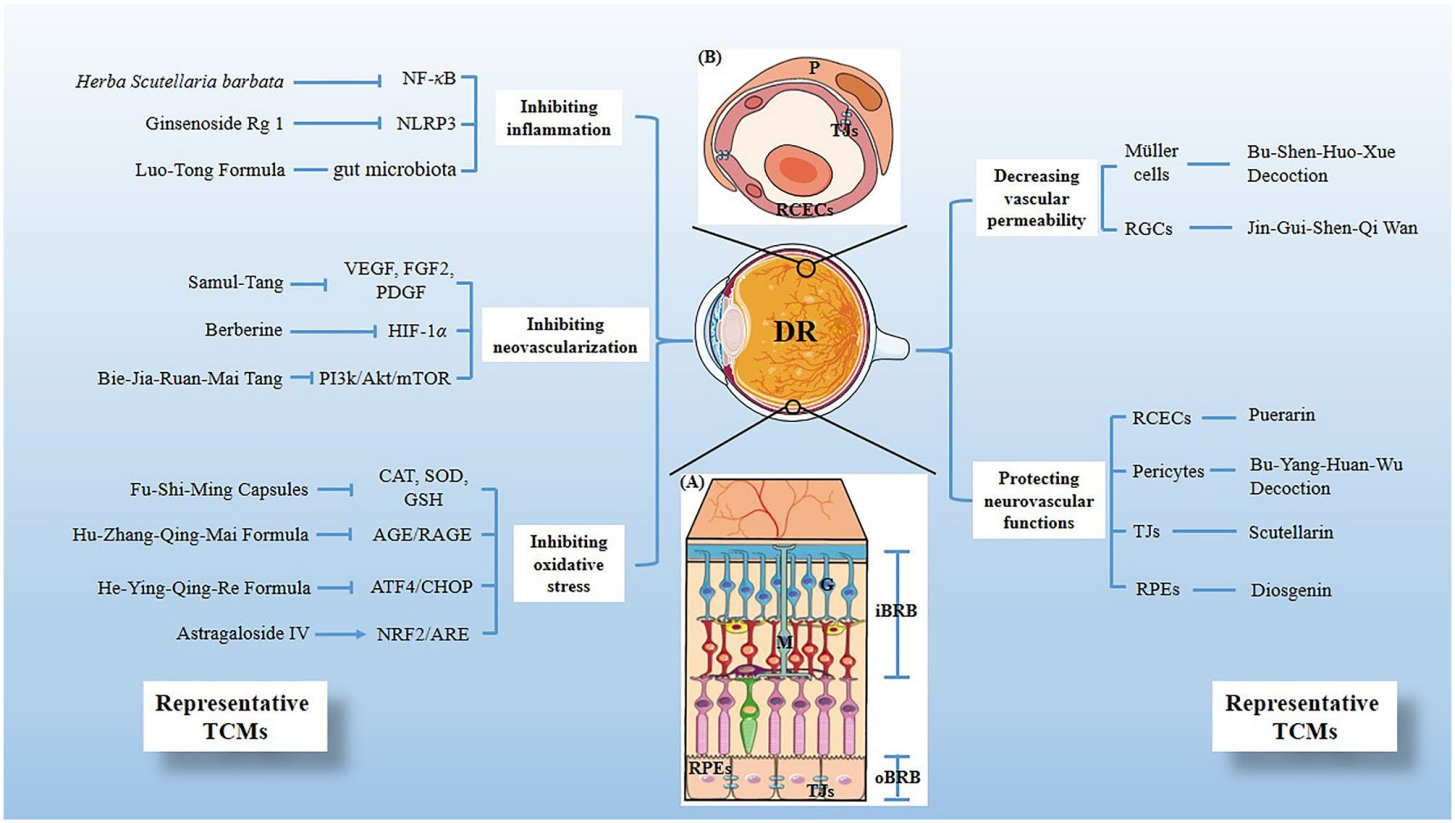
Scheme illustrating the mechanisms of traditional Chinese medicines (TCMs) on diabetic retinopathy and its representative TCMs. (A) The BRB consists of two parts, the inner BRB (iBRB) and the outer BRB (oBRB) that together regulate the exchange of substances between the retina and the systemic circulation. In addition, the components involved in the therapeutic mechanism of DR are annotated in the figure, including RGCs (G), Müller cells (M), RPEs and TJs. (B) Magnified cross-sectional view of the retinal vessel mainly constituents of the iBRB, including RCECs, Pericyte (P) and TJs.

During the past several decades, a substantial body of research has been conducted on the underlying mechanisms of TCMs treatments for DR. These investigations focused on various therapeutic approaches, including inhibiting oxidative stress, reducing inflammation and neovascularization, protecting neurovascular functions, and decreasing vascular permeability. Figure 1 provides a summary of these mechanisms and also lists some representative traditional Chinese medicines (Figure 1). Understanding these mechanisms represents a critical breakthrough in elucidating the pathogenesis of DR, contributing significantly to the development and refinement of TCM-based treatments.

#### Inhibiting oxidative stress

Oxidative stress is a crucial contributor to the pathogenesis of DR, leading to structural and functional changes in retinal microvessels (Madsen-Bouterse and Kowluru 2008). It represents an imbalance between the production of free radicals and reactive metabolites, or reactive oxygen species (ROS), and their elimination by protective mechanisms (Li, Miao, et al. 2017). TCMs can directly decrease oxidation markers or modulate the AGE/RAGE, ATF4/CHOP, and NRF2/ARE signaling pathways to inhibit oxidative stress.

Antioxidative enzymes such as glutathione peroxidase (GSH-Px), superoxide dismutase (SOD), and catalase (CAT) effectively reduce oxidative stress. Fu-Shi-Ming Capsules (FSM), manufactured by Xi’an Lejian Biological Technology Co., Ltd., contain five active compounds and have shown potential in anti-oxidative stress therapy. In DR rats treated with FSM, there was an improvement in serum anti-oxidative enzymes (GSH-Px, SOD, CAT), highlighting the ability of FSM against DR by enhancing antioxidant activity (He et al. 2019). Similarly, Naringin, a flavonoid found in some Chinese herbs and citrus fruits (Mouly et al. 1993), was as effective as FSM in raising the levels and activities of GSH, SOD, and CAT in DR rats (Liu et al. 2017).

AGEs, a wide range of proteins, activate various downstream pathways and maintain oxidative stress in vascular tissues when bound to RAGE, their receptor (Xu et al. 2018). Research has shown that the Hu-Zhang-Qing-Mai Formulation (HZQMF), with quercetin as the primary active ingredient, reduced H_2_O_2_ damage effectively by inhibiting apoptosis-related genes in the AGE/RAGE pathway (Wu et al. 2023). Similarly, the He-Ying-Qing-Re Formula (HF) decreased AGE levels in the retina and prevented RAGE expression induced by AGEs (Wang et al. 2015). Berberine (BBR) also positively affected blood glucose levels and AGE formation while inhibiting the AGE/RAGE signaling pathway in the retina (Wang, Wang, et al. 2021).

Endoplasmic reticulum stress (ERS), with ATF4/CHOP-mediated cell death, is a significant mechanism in diabetes-induced oxidative damage. CHOP, a pro-apoptotic transcriptional effector, increases ROS production and enhances apoptosis response feedback, and ATF4 is a critical upstream transcriptional factor (Han et al. 2013). HF treatment in diabetic mice showed a reversal of RGC depletion through ROS elimination, and reduction of ATF4/CHOP-mediated ERS (e.g., cleaved caspase-3 and phosphorylated-p38 MAPK). Additionally, it enhanced the expression of anti-apoptotic factors, such as Bcl-2 and Bcl-x (Zhang, Xu, Tan, et al. 2018).

The NRF2/ARE signaling pathway is an effective means of alleviating oxidative stress. Activation of the NRF2 leads to its binding to the antioxidant response element (ARE), thereby initiating the transcription of downstream genes. This process improves cellular antioxidant and damage repair capabilities (Tu et al. 2019). Both the ferroptosis inhibitor and amygdalin, extracted from *Prunus armeniaca* L. (Rosaceae), relieved ferroptosis caused by oxidative stress, with the effect of amygdalin being dependent on NRF2 pathway activation (Li et al. 2023). Astragaloside IV also protected the retinas of DR rats by activating the NRF2 pathway to reduce oxidative stress injury (Yu et al. 2020).

#### Relieving inflammation

Inflammation plays a crucial role in DR development and progression. In fact, diabetic retinopathy can be considered a condition of low-grade chronic inflammation (Rangasamy et al. 2012). Numerous studies have indicated that TCM has a potent anti-inflammatory potential. This is achieved through mechanisms such as inhibiting the NF-κB signaling pathway, preventing the formation of the NLRP3 inflammasome, and regulating the gut microbiota.

NF-κB is a key regulator of pro-inflammatory gene transcription and plays a critical role in managing retinal inflammation during DR progression. The classical NF-κB signaling pathway is activated as follows: IκB kinase (IKK) activation leads to the translocation of p65 NF-κB dimers from the cytoplasm to the nucleus, where they bind to DNA and promote transcription (Polley et al. 2016). Activation of the NF-κB pathway regulates the expression of pro-inflammatory mediators like TNF-α, IL-1β, ICAM-1, VCAM-1, and various chemokines. This cascade further promotes an inflammatory response, leading to retinal endothelial injury and microvascular leakage in diabetic retinas (Rangasamy et al. 2012).

An aqueous extract of *Astragalus membranaceus* Bunge (Fabaceae), *Angelica sinensis* (Oliv.) Diels (Apiaceae), and *Panax notoginseng* (Burkill) F.H.Chen ex C.Y. Wu & K.M. Feng, (Araliaceae) reduced the expression of inflammatory biomarkers such as IL-1β, IL-6, TNF-α, NF-κB p65, MCP-1, ICAM-1, and VCAM-1 in diabetic rats (Gao et al. 2013). Elema-1,3,7(11),8-tetraen-8,12-lactam (Ele) which was isolated from *Curcuma wenyujin* Y.H. Chen & C. Ling (Zingiberaceae), downregulated the mRNA levels of TNF-α and ICAM-1 in TNF-α-induced human umbilical vein endothelial cells (HUVECs). This effect may be mediated by inhibiting the IKK/NF-κB signaling pathway (Cai et al. 2024). Furthermore, *Panax notoginseng* saponins (PNS), the primary active constituent of *Panax notoginseng*, played a crucial role in decreasing the number of acellular capillaries by exerting anti-inflammatory effects through the NF-κB pathway (Wang, Sun, et al. 2023). Similarly, *Rhodiola sachalinensis* A. Boriss (Crassulaceae) (RS) (Zhao et al. 2013) and *Scutellaria barbata* D. Don (Lamiaceae) (SE) (Mei et al. 2017) were effective in reducing inflammatory mediators of the NF-κB pathway.

The NLRP3 inflammasome, a cell membrane multimeric complex, mediates downstream cellular inflammatory responses and triggers pyroptosis after injury detection (Yang, Jiang, et al. 2023). Toll-like receptor 4 (TLR4), a member of the TLR family, activates various transcription factors, including NF-κB. The NLRP3 inflammasome also stimulates the TLR4/NF-κB axis, linking it to the NF-κB signaling pathway and increasing the expression of TNF-α (Eissa et al. 2021). A preclinical study which treated type 2 DR mice with ginsenoside Rg1 showed that ginsenoside Rg1 reduced the expression of NLRP3, p-NF-κB, caspase-1, and IL-1β in the retina. This suggested that ginsenoside Rg1 alleviated DR by inhibiting the pathways involved in the NLRP3 inflammasome (Li et al. 2022a). Additionally, nimbolide, a terpenoid compound found in the neem plant [*Azadirachta indica* A, Juss. *(*Meliaceae)], was administered for 60 days and directly inhibited the TLR4/NF-κB signaling pathway (Shu et al. 2021).

The Luo-Tong Formula (LTF) lowered the levels of seven inflammatory-related cytokines, resulting in an anti-inflammatory effect. Intriguingly, in this research, certain gut microbiota in DR rats were significantly correlated with inflammatory factors. This finding led researchers to propose the concept of the ‘gut microbiota inflammatory visual network axis’ (Di et al. 2022). The association of this axis with chronic inflammatory states has been increasingly demonstrated in the development of DR.

#### Improving neurovascular dysfunction

Improving neurovascular dysfunction presents a potential therapeutic strategy to prevent diseases linked to DR. This approach is based on the emerging concept that neurodegeneration is an early event in DR development (Simó and Hernández 2023). The retinal neurovascular unit (NVU) is essential for maintaining the stability of the retinal environment and supporting retinal metabolism. NVU comprises various neural cell types (e.g., ganglion cells, amacrine cells, horizontal, and bipolar cells), glia (Müller cells and astrocytes), professional immune cells, and vascular cells (Yang, Huang, et al. 2023). This section focused on protecting retinal ganglion cells (RGCs) and Müller cells, as well as preventing glutamate accumulation, as treatment strategies for DR.

First, the protection of RGCs, an optional therapeutic measure, can delay disease onset caused by RGCs injury in the DR process. TCM has been found to be effective in preserving RGC numbers. For example, diosgenin (DG), a natural steroidal sapogenin, increased Bcl-2 expression while decreasing cleaved caspase-3, thus playing a central role in inhibiting RGCs apoptosis (Liao et al. 2023). Jin-Gui-Shen-Qi Wan (JGSQ), first recorded in ‘Essentials from the Golden Cabinet’ and used for thousands of years in China since the Eastern Han dynasty (Hu et al. 2021), reduced RGC apoptosis compared to the model group. This reduction was achieved by increasing the expression of p-AKT and decreasing the hypoxia-inducible factor (HIF)-1α (Liang et al. 2023). Additionally, Pu-Ren Dan, as mentioned above, has been demonstrated to be an effective treatment for DR, as evidenced by both clinical effects and the protection of RGCs (Dai et al. 2016). Furthermore, Müller cells, which serve as the retinal nutrition and structural maintenance unit and the largest gliocyte in the retina, play a crucial role in DR pathogenesis (McDowell et al. 2018). Gypenoside XVII (Gyp-17) protected Müller cells in early-stage DR mice by upregulating Bcl-2 and ATG-5 expression and downregulating Bax and P62 protein expression in Müller cells. These results revealed that Gyp-17 prevented early DR by decreasing apoptosis and increasing autophagy in Müller cells, contributing to its neuroprotective effects (Luo et al. 2021).

Glutamate, the major excitatory neurotransmitter in the central nervous system, including the retina, has comprehensive physiological functions such as neurotransmission. However, excessive glutamate amounts or activity can be toxic to neurons, implicating glutamate excitotoxicity in several diseases. Fortunately, glutamate can be converted to glutamine by glutamine synthetase, expressed primarily in Müller cells, to protect neurons from glutamate-induced toxicity (Taylor et al. 2003). The effects of Bu-Shen-Huo-Xue Decoction (BSHX), composed of *Salvia miltiorrhiza* Bunge (Lamiaceae) and *Rehmannia glutinosa (Garetn.) DC.* (Orobanchaceae), on RGCs, Müller cells, and glutamine synthetase co-cultured *in vitro* under high glucose and hypoxia conditions were investigated. BSHX has been proven to play a neuroprotective role by increasing GS activity, enhancing glutamate intake, and reducing glutamate release in DR treatment (Ma et al. 2014, 2015). Activation of sigma-1R inhibited glutamate-induced apoptosis in retinal neurons (Senda et al. 1998). Mi-Meng-Hua Decoction promoted RGC-5 cell proliferation and inhibited apoptosis by regulating neurotrophic factors and activating sigma-1R (Wu et al. 2018).

TCM can regulate gene expression to exert neuroprotective effects. Shuang-Dan-Ming-Mu Capsules, manufactured by Beijing Qihuang Pharmaceutical Co., Ltd. (Batch number: Z20080062), improved retinal morphology, effectively restored the number of cells in the ganglion cell layer, and reduced apoptosis in all retinal layers by regulating the expression of miR-450b-5p, miR-1249, and miR-155-5p (Chen et al. 2019; Li, Yang, et al. 2022). Additionally, the mechanisms of the Huo-Xue-Jie-Du Formula (HXJDF) involved regulating microRNA levels (e.g., miR-423-5p, miR-341, miR-3562, miR-3588, miR-500-3p, miR-3099) to restore RGC numbers and inhibit retinal cell apoptosis (Li et al. 2021).

#### Reducing vascular permeability

The structural integrity of the BRB is crucial to maintain vascular permeability and visual function. It is responsible not only for maintaining homeostasis in the retinal microenvironment by regulating the exchange of molecules between the systemic circulation and the retina but also for facilitating nutrient and oxygen delivery while preventing blood-borne toxins from entering. The BRB comprises an inner blood-retinal barrier (iBRB), formed by tight junctions (TJs) between retinal capillary endothelial cells (RCECs) and surrounding pericytes, and an outer blood-retinal barrier (oBRB), formed by retinal pigment epithelial (RPE) cells (Cunha-Vaz et al. 2011). The maintenance of permeability is highly dependent on the integrity of these TJs. Therefore, protecting RCECs, RPE cells, and regulating TJs are beneficial in reducing the increased vascular permeability caused by disease.

Tight junctions, such as ZO-1, occludin, claudin-1, claudin-5, and claudin-19, are crucial to the functionality of the BRB, with its physiological functions relying mainly on the integrity of these TJs between cells (Díaz-Coránguez et al. 2017; Erickson et al. 2007). The whole dried plant of *Scutellaria barbata* D. Don. (Lamiaceae) (SE) has been shown to attenuate the development of DR by restoring the decreased expression of TJs, including claudin-1 and claudin-19 (Mei et al. 2017). Similarly, a study on the ethanol extract of *Dendrobium chrysotoxum* Lindl. (Orchidaceae) (DC) in DR revealed that DC prevented the decrease of both proteins and mRNA levels in TJs, such as occludin and claudin-1 (Yu, Gong, et al. 2015). Moreover, scutellarin (SC) was found to reverse increased Evan’s blue leakage and decreased expression of claudin-1 and claudin-19 in retinas by activating Nrf2, a finding validated using Nrf2 knockout diabetic mice. This demonstrated the critical role of Nrf2 in SC-provided protection against BRB injury during DR development (Mei et al. 2019).

Dysfunctional signaling, abnormal metabolism, and pathological states can disrupt the balance between pericytes and RCECs, the two main cellular components of iBRB, leading to the breakdown of BRB and other microangiopathies (Huang 2020). Additionally, diabetic endothelial dysfunction, such as leukostasis and cell apoptosis, plays a central role in initial changes in BRB (Cunha-Vaz 1983). TCMs have been effective in protecting the function and integrity of RCECs in iBRB. For example, Fu-Fang-Xue-Shuan-Tong (FXST) protected retinal vascular endothelial cells under high glucose conditions. In particular, the disappearance of improved function in retinal vascular endothelial cells was observed when adding Yes-associated protein inhibitors to the FXST treatment group, suggesting that the Yes-associated protein was a target of FXST (Choi et al. 2015; Xing et al. 2019). VEGF was important not only in angiogenesis but also in the pathogenesis of DR-related hyperpermeability, which was suppressed by inhibiting ROCK (Sun et al. 2006). The effects of *Lycium barbarum* L. (Solanoideae) polysaccharides (LBP) on retinal abnormalities in STZ-induced diabetic rats and choroidal-retinal endothelial cells showed that LBP inhibited angiogenesis and reversed upregulated ROCK1 in diabetic rats, indicating the protective effects of LBP on the BRB by targeting the Rho/ROCK1 signaling pathway (Wang, Yao, et al. 2019). Furthermore, ginsenoside Rd (Rd) has been identified as a potential medicine for DR treatment by inhibiting oxidative stress-mediated mitochondrial dysfunction (Tang et al. 2022). Puerarin exhibited protective effects on retinal endothelial cells in rats by inhibiting IL-1β-mediated inflammation (Zhu et al. 2014). Additionally, TCMs have shown effectiveness in pericytes’ protection. For example, an extract of *Astragalus membranaceus* inhibited apoptosis and improved the viability of bovine retinal capillary pericytes cultured in high glucose *in vitro* (Yang and Duan 2007). Bu-Yang-Huan-Wu Decoction improved STZ-induced retinal microangiopathy in rats and inhibited pericytes’ apoptosis in the retinal microvasculature (Liu et al. 2013).

RPE cells, which maintain the structural integrity of the retina and choriocapillaris by secreting molecules such as pigment epithelial-derived factor (PEDF), are explicitly related to oBRB. PEDF competes for the VEGF receptor, reducing VEGF levels and new blood vessel formation. A decrease in PEDF levels also accelerates apoptosis and dysfunction of pericytes in the retina (Li, Li, et al. 2013). Therefore, protecting the physiological function of RPE cells offers a new approach to DR treatment. *Scoparia dulcis* L. (Plantaginaceae) extract protected ARPE-19 cells from apoptosis by inhibiting oxidative and inflammatory stress through the Akt/Nrf2/HO-1 pathway (Lin et al. 2023). Similarly, diosgenin increased the viability of ARPE-19 cells induced by high glucose, benefiting from activating the AMPK/Nrf2/HO-1 pathway and reducing the inflammatory response and oxidative stress in ARPE-19 cells (Hao and Gao 2022).

#### Inhibiting neovascularization

PDR, characterized by the development of new blood vessels, represents the most advanced stage of diabetic eye disease in both type 1 and type 2 diabetes. It leads to central and peripheral vision loss when these fragile new vessels bleed into the vitreous (Crabtree and Chang 2021). Significant advances have been made in TCMs to inhibit neovascularization, and this section focused on related mechanisms, such as reducing angiogenic molecules and inhibiting the HIF-1α and phosphatidylinositol-3 kinase (PI3k)/Akt/mTOR signaling pathways.

Under physiological conditions, neovascularization may be triggered by specific angiogenic molecules such as VEGF, platelet-derived growth factor (PDGF), and fibroblast growth factor 2 (FGF2). Inhibiting neovascularization can be achieved by directly decreasing these pro-angiogenic factors. VEGF is a key regulator of angiogenesis, and anti-VEGF therapy remains a recommended clinical treatment for DR (Wong et al. 2016). Samul-Tang (SMT) and Guibi-Tang (GBT), commonly used in East Asia, have been shown to reduce VEGF levels (Lee, Kim, et al. 2015; Lee, Lee, et al. 2015). FGF2, akin to VEGF, is a potent pro-angiogenic signaling molecule, and these two factors together might produce a synergistic angiogenic response (Asahara et al. 1995). GBT also exhibited inhibitory effects on angiogenesis by downregulating FGF2 compared to the model group, suggesting the impact of GBT in reducing the central non-perfusion area and the formation of retinal neovascular tufts occurs *via* inhibition of both VEGF and FGF2 (Lee, Lee, et al. 2015). PDGF-BB is another pro-angiogenic factor (Heldin and Westermark 1999) which is implicated in the development of proliferative retinopathies when PDGF activity is excessive (Lefevere et al. 2022). Sipjeondaebo-Tang (SDT), a traditional formula used widely for a long time, has been shown to reduce the expression of PDGF-AB/BB compared to the model group. It also blocked the binding of PDGF-BB to its receptor in a ligand-binding assay *in vitro* (Lee et al. 2014).

HIF-1 can activate VEGF and elevate VEGF mRNA levels by inhibiting degradation during hypoxic conditions, leading to neovascularization (Choudhry and Harris 2018; Damert et al. 1997). *Berberis dictyophylla* Franch. (Berberideae), a well-known Tibetan herb with a wide range of pharmacological functions, has been frequently used in treating DM. Some detrimental alterations in the retina, such as hyperplasia and vessel dilation, were dramatically reversed by Xiao Bopi (XBP), derived from the stem bark of *Berberis dictyophylla*. This effect was achieved through the mechanism of suppressing the HIF-1α/VEGF/DLL-4/Notch-1 pathway (Ai et al. 2022). Interestingly, insulin has been found to increase the incidence of diabetic retinopathy by inducing VEGF and HIF-1α expression, particularly in retinal endothelial cells (Wat et al. 2016). Berberine, a natural bioactive alkaloid, has shown potential as a potent inhibitor of insulin-induced VEGF and HIF-1α overexpression. This inhibitory effect can be abolished by reactivating Akt/mTOR activity in HRECs (Wang, Zhang, et al. 2021).

Akt is a crucial signaling center for cell growth and survival. It not only counteracts apoptosis by downregulating pro-apoptotic signals but also promotes protein synthesis and cell growth by activating mTOR complex 1 (mTORC1) induced by PI3K (Li and Wang 2014). Bie-Jia-Ruan-Mai Tang, a traditional compound, has been shown to inhibit the growth of acellular capillaries in the retina and the proliferation of human retinal capillary endothelial cells (HRCECs) caused by high glucose. The mechanism behind this effect was the suppression of the PI3K/AKT signaling pathway (Liu et al. 2022).

## Discussion

DR is one of the common microvascular complications among diabetic patients and remains a leading cause of visual loss in the working-age population. The ‘Chinese Guidelines for the Clinical Diagnosis and Treatment of Diabetic Retinopathy (2022)’ categorized DR into PDR and NPDR based on the disease’s progression. Additionally, according to TCM syndrome differentiation, DR was classified as *Qi-Yin* deficiency, and some cases presented a combination of *Qi-Yin* deficiency and meridian stasis syndrome (Zhao et al. 2017).

This review synthesized the clinical benefits of TCM in treating DR, including traditional empirical formulas, patent medicines, and isolated phytochemicals. Chinese medicines have been shown to reduce the number of micro-aneurysms (Chen 2023; Dai et al. 2020; Lian et al. 2015), decrease the area of retinal hemorrhage and exudation (Chen 2023; Dai et al. 2020; Huo et al. 2022; Lian et al. 2015), improve visual acuity (Sun and Jiang 2013; Wang, Zhang, et al. 2023), and improve hemorheological indicators (Li, Feng, et al. 2017). Furthermore, they have been shown to reduce the mean defect area and capillary nonperfusion area (Luo et al. 2015) in relevant clinical research. TCM treatments for DR target not only the lesion site but also adopt a holistic approach (Liang et al. 2020), offering the unique advantage of reducing TCM syndrome scores (Huo et al. 2022; Wang, Zhang, et al. 2023; Zhang et al. 2020).

We summarized the mechanisms of traditional Chinese empirical formulas, traditional Chinese patent medicines, and isolated phytochemicals in treating DR. These mechanisms include: (1) inhibiting oxidative stress by reducing markers of oxidation levels (CAT, SOD, GSH), inhibiting the AGE/RAGE signaling pathway, the ATF4/CHOP signaling pathway, and activating the NRF2/ARE signaling pathway; (2) relieving inflammation through the inhibition of the NF-κB signaling pathway and the formation of NLRP3 inflammasomes, and the regulation of gut microbiota; (3) inhibiting neovascularization by reducing angiogenic molecules (VEGF, FGF2, PDGF), and inhibiting the HIF-1α and the PI3k/Akt/mTOR signaling pathways; (4) improving neurovascular dysfunction by protecting Müller cells and retinal ganglion cells (RGCs), and inhibiting glutamate accumulation; (5) reducing vascular permeability, focusing on the protection of the blood-retinal barrier, involving the inner BRB – pericytes and retinal capillary endothelial cells, the outer BRB – retinal pigment epithelium cells, and tight junctions.

Considering the long-term development of TCM in the prevention and treatment of DR, significant progress has been made in recent years. However, several aspects warrant further consideration. First, while TCM offers the advantage of personalized therapy, its effectiveness is often subject to significant subjective variability. This is because syndrome differentiation and disease staging in TCM require the cooperation of clinicians, heavily relying on the doctor’s judgment (Li 2007). Concurrently, the advancement in the study of new analytical techniques for biomarkers related to DR accelerates progress in the field, enhancing our understanding of the disease (Jenkins et al. 2015). This suggests that more emphasis should be placed on researching the underlying mechanisms and integrating TCM with new techniques and methods, in addition to exploring the efficacy of TCM in treating DR. This integration could potentially improve the prevention of vision loss in patients with diabetic retinopathy. Second, most TCMs reviewed here focus primarily on the NPDR stage, as indicated by our analysis (Wang, Wu, et al. 2023). During the PDR stage, TCM is often only used as an adjunct (Wang, He, et al. 2019). Therefore, it is crucial to further explore the potential of TCM in treating the PDR stage. Understanding how to correctly use traditional Chinese medicine at the appropriate stage of the disease is also essential.

## Conclusions

TCMs are characterized by large sources, low safety risks, and various pharmacological activities. Many studies confirmed that TCMs had high values in the intervention of DR disease. The mechanisms include inhibiting oxidative stress, relieving inflammation, inhibiting neovascularization, improving neurovascular dysfunction, and reducing vascular permeability. Therefore, it is feasible to treat DR with TCMs, and research on the exact mechanism of TCM is a possible direction in the future.

## Data Availability

The data are available from the corresponding author upon reasonable request.
